# Supplementary material for: Changes in unmet need for family planning among married women of reproductive age in Nigeria: A multilevel analysis of a ten-year DHS wave
Source: PLoS One. 2024 Aug 2;19(8):e0306768. doi: 10.1371/journal.pone.0306768 (PMC11296638; doi:10.1371/journal.pone.0306768)
Supplement: Attachment — Submitted filename: Comments to the authors.docx [file pone.0306768.s001.docx]

Dear editorial team,

Thank you for inviting me to review this fascinating title on the global agenda of women's health. Dear authors, I appreciate your efforts in coming up with an interesting title, although it does require substantial revision. Below, I have provided section-by-section review comments that I highly recommend you address point by point.

**General comments**

**Introduction:**

The introduction lacks clarity and fails to provide a strong background for the study. Please consider rephrasing it to clearly outline the purpose and significance of the research.

**Literature Review:**

The literature review is comprehensive but lacks recent references. Please update it with more current sources to ensure the study is up to date.

**Methodology:**

The methodology section needs further elaboration. Please provide more details on the sample size, data collection methods, and statistical analysis techniques used.

**Results:**

The results section is well-presented and provides valuable insights. However, it would be beneficial to include more graphical representations, such as charts or tables, to enhance the clarity of the findings.

**Discussion:**

The discussion section needs to be more focused and structured. Please organize the key findings and relate them back to the research objectives. Additionally, consider discussing the limitations of the study and suggesting areas for future research.

**Conclusion:**

The conclusion should be more concise and summarize the main findings of the study. Avoid introducing new information in this section.

**Language and Writing Style:**

The overall language and writing style of the manuscript need improvement. Please ensure that the text is clear, concise, and free from grammatical errors. Consider seeking assistance from a professional editor to enhance the readability of the paper.

**Specific comments**

Abstract

1. Methods and materials could revised as methods only

2. You study about unmet need for family planning while you concluded about family planning, since unmet need isnot equal with family planning

Introduction

1. It is good while some grammatical errors there and

2. There is also study conducted on unmet need for family planning while you did not mentioned it (Solanke et al.2022,adebowale et al,2023 based on 2018 DHS…)

3. Since DHS data is open access you did not assure it is whether conducted or not similar study

4. There is study conducted based on Nigeria DHS 2018 which is recent data and important for policy and program, if this study conducted what is the important of studying on same title?

5. Trends of unmet need conducted by Oginni et al. 2015(based on DHS 2008-2013) and Adebowale et al. 2023( based on 2018 DHS) if so what new finding is added this study?

Methods

1. How to manage missing values

2. Detailed analysis of the data not described

3. Description of independent variables mention in tables

4. What about clustering effect

5. Random effect and fixed effect, is two equally used how?

Results

1. What does mean Univariate Results? In table descriptive statistics/sociodemographic characteristic

2. Missing value not reported, why? Since this is secondary data missed data is expected

3. You use fixed effect model, how? This is country level data so how fixed effect model used?

4. The ICC value decreased what does mean?

Discussion

1. The implication not included please include in discussion

2. Comparison with previous literatures not well discussed

3. Include national level reports of unmet need during discussion

4. Religion is one of the factors, how did you recommended for programs and policy makers

5. You calculated Odds ratio but on discussion it says there is a positive, how?

Limitation

1. What about missed value, using secondary data, behavioral factors?
