# Supplementary material for: Changes in unmet need for family planning among married women of reproductive age in Nigeria: A multilevel analysis of a ten-year DHS wave
Source: PLoS One. 2024 Aug 2;19(8):e0306768. doi: 10.1371/journal.pone.0306768 (PMC11296638; doi:10.1371/journal.pone.0306768)
Supplement: Attachment — Submitted filename: RESPONSES TO REVIEWERS.docx [file pone.0306768.s002.docx]

**EDITORIAL DECISION:** Revision required [PONE-D-24-04879]

Dear Editorial Team,

PLOS ONE,

Thank you for giving us the opportunity to submit a revised draft of our manuscript titled “Changes in unmet need for family planning among women of reproductive age in Nigeria: a multilevel analysis of a ten-year DHS wave” for publication in the PLOS ONE. We appreciate the time and effort that you and the reviewers have dedicated to improve the readability of the paper as well as providing valuable improvements to our paper. We have incorporated the suggestions made by the Editorial Team/reviewers in the revised manuscript file labelled “Response to Reviewers”, “Revised Manuscript with Track Changes” and “Manuscript”. We hereby provide a point-by-point response to the Editorial Team’ comments and concerns.

**Major Corrections**

**REVIEWER #1:**

**Reviewer:** The paper falls short of its objectives. It does very little to show the changes across the period in unmet need for family planning. While it shows progress in unmet need using a trend analysis, this does not delve into the changes and potentials that could be responsible for these changes

**Response:** Thank you for the observation. Changes across the period in unmet need for family planning and factors responsible for these changes have been included in the manuscript.

**Reviewer:** The researchers however, investigated the factors that could influence the unmet need for family planning. They start by making an effort to use Chi-square from bivariate analysis to make a statement on the observation. However, this statement is confusing. ‘Table 2 indicate that age, region, education, wealth quantile, religion, parity of respondents, sex of household head, partner education, experience of child’s death and community literacy are all significant predictors of unmet needs for family planning among women of reproductive age between 2008 and 2018 with p values < 0.000.’ The only thing Chi-square would tell us is that the proportions in each group being compared are different. The values could be swinging and going up and down and this does not tell in any way if they are predictors of unmet need for family planning

**Response:** Thank you for the observation. The chi-square test has been recomputed and the p- values for each category has been provided to show that the proportions in each group being compared are different.

**Reviewer:** The researchers note that their study is a cross-sectional study. It has been suggested in the annotated document that the researchers should recast that the study used secondary data that had been collected through a cross-sectional survey. They didn’t design the study and cannot now change the design.

**Response:** Thank you for the observation. It has been corrected to read “the study used secondary data from the three rounds of the Nigeria Demographic and Health Survey (NDHS) conducted in 2008, 2013, and 2018 that had been collected through a cross-sectional survey design.”

**Reviewer:** For factors that are associated with unmet need for family planning, the researchers used an appropriate method. However, the study is noted to be about the changes in unmet need which this investigation failed to elaborate on. There are several studies that have investigated the factors influencing unmet need for family planning and one does not immediately see the added value that this analysis brings to the table as these same predictors have been identified in previous studies.

**Response:** Thank you for the observation. The study has explained the changes in unmet need and potentials that could be responsible for these changes

**MINOR CORRECTIONS**

**Introduction**

**Reviewer:** This statement: ‘Promotion of use of modern contraception has been mainly advocated by non-governmental, international and community-based organisations.’ – Would be good to give a reference for such statement. Otherwise, it is your opinion. I am certain several governments have made policy commitments towards control of population explosion. China is an extreme one. So, your statement might not be absolutely correct.

**Response:** Thank you for the comments. The paragraph has been revised and references provided to support the statements. Emphasis is made on different interventions designed and implemented by the Nigerian government with verifiable references. The paragraph is now better in coherence and narrative flow of thoughts.

**Reviewer:** Meanwhile, government interventions from international organizations and other stakeholders aimed at reducing high unmet need for family planning among women by making family planning methods available and accessible have yielded little progress in Nigeria [13, 14]. - Can you explain better what you mean by this statement? You had stated earlier that government wasn't doing much. You might want to revise that statement.

**Response:** The contradictory statements in the introductory paragraph have been replaced by more logical arguments and synthesis of thoughts.

**Reviewer:** For instance, in Ghana the importance of studies on unmet need for improving family planning uptake was established [14, 15, 16]. – Established what? In a study, a prevalence of 30% was found among married Ghanaian women [15]. – Sentence can be better cast. 30% of what?

**Response:** This sentence has been rephrased for better coherence and readability.

**Sample Design**

**Reviewer: (line 122-124).** A cross-sectional research study design using secondary data from three rounds of the Nigeria Demographic and Health Survey (NDHS) conducted in 2008, 2013, and 2018 was employed in this study. The women of reproductive age (15–49 years) in the surveys were asked retrospective questions covering the 5 years preceding the survey. - Did you use a cross sectional study design? Or was the secondary data you used collected through cross-sectional means? Your statement is as though you designed the study. Please revise appropriately.

**Response:** Thank you for the observation. It has been revised appropriately to read “The study used secondary data from three rounds of the Nigeria Demographic and Health Survey (NDHS) conducted in 2008, 2013, and 2018 that had been collected through a cross-sectional survey.

**Exclusion and Inclusion**

**Reviewer:** (Line 181) The revised definition of unmet need for family planning [17, 25] was applied to estimate total unmet need (spacing and limiting). - Good to explicitly provide this definition here. ‘Women who were not using contraception, who were pregnant or amenorrheic, or whose pregnancy or birth was mistimed or unwanted;’ – Between amenorrhoeic and whose should have been an ‘AND’ operator from the schema diagram that you provided.

**Response:** Thank you for the observation. The ‘AND’ operator has been inserted while “OR” has been deleted. It now reads. ‘Women who were not using contraception, who were pregnant or amenorrheic, and whose pregnancy or birth was mistimed or unwanted;’

Explanatory variable

**Reviewer:** (line 207) The individual level variables were as follows: age – This should be age group

**Response:** Thank you for the observation. However, respondent’s age was only categories and presented in group. The variable name should be age as earlier presented.

**Statistical Analysis**

**Reviewer: (line 222-223).** These three sets of DHS datasets were used separately to examine the regional trends in unmet need for family planning, while the data were pooled to examine the contextual correlates of unmet family planning needs in Nigeria. - Why pool the data if you believe it should be changing from year to year? Or what is the rationale for the trend analysis and the title of ‘Change’?

**Response:** Thank you for the observation. It has been revised to read “The pooled data were used to examine changes and contextual determinants of unmet need for family planning in Nigeria.

*******************************************************************************

**REVIEWER 2:**

GENERAL COMMENTS

**Introduction:**

**Reviewer:** The introduction lacks clarity and fails to provide a strong background for the study. Please consider rephrasing it to clearly outline the purpose and significance of the research.

**Response:** The introduction is now engaging, narrowed and succinct. There is a clear focus on changes and contextual determinants of unmet need for family planning. The other components of the introduction were delineated to improve readability and organisation. This has made the introduction more convincing and logical. The purpose and exact argument of this study is now clear. The write-up focuses and delves into relevant ideas. The introduction addresses the crux of the matter, changes in unmet need for family planning, its implications and how contextual factors contributes to it. The introduction is now structured based and written based on a framework called the deficiency model of scholarly introduction in social sciences. We identified the research problem, reviewed existing studies, identified gaps in previous studies and how our study intends to address the gaps. We ended the introductory section by stating the purpose of the study.

**Literature Review:**

**Reviewer:** The literature review is comprehensive but lacks recent references. Please update it with more current sources to ensure the study is up to date.

**Response:** Recent literature has been cited, argued and synthesised.

**Methodology:**

**Reviewer:** The methodology section needs further elaboration. Please provide more details on the sample size, data collection methods, and statistical analysis techniques used.

**Response:** Thank you for the observation. The information on the sample size and data collection methods were provided under the title “Data source, study population and sample size”. The statistical analysis techniques used was also provided under the title “Statistical analysis”. However, as suggested by Reviewer #3, we don’t need to add more details than what we already had in this manuscript since the data is a secondary data which has been reported elsewhere. Thus, it has been corrected as suggested by Reviwer #3.

RESULTS:

**Reviewer:** The results section is well-presented and provides valuable insights. However, it would be beneficial to include more graphical representations, such as charts or tables, to enhance the clarity of the findings.

**Response:** Thank you for the observation. However, the results have been presented using tables or charts. Most journals have specified number of charts or graphical representations.

**Discussion:**

**Reviewer:** The discussion section needs to be more focused and structured. Please organize the key findings and relate them back to the research objectives. Additionally, consider discussing the limitations of the study and suggesting areas for future research.

**Response:** Thank you for the feedback. The discussion section has been duly addressed with focus on the objectives of the study. More so, the limitation of the study has been discussed and addressed. Area for further research has now been addressed under the conclusion section.

**Conclusion:**

**Reviewer:** The conclusion should be more concise and summarize the main findings of the study. Avoid introducing new information in this section.

**Response:** The conclusion was as concise as possible and gave a detailed summary of the findings of the study. No new information was introduced into the section that contradicts the purpose of the section. This now a clear philosophic stance about the overall message of the study.

**Language and Writing Style:**

**Reviewer:** The overall language and writing style of the manuscript need improvement. Please ensure that the text is clear, concise, and free from grammatical errors. Consider seeking assistance from a professional editor to enhance the readability of the paper.

**Response:** Thank you for the suggestion. The manuscript has been revised and edited. Thus, all grammatical and typographical errors have been addressed.

**Specific comments**

**Abstract (line 11- 46)**

**Reviewer:** 1. (Line 19) Methods and materials could revised as methods only

**Response:** Thank you for the observation. It has been corrected to read Methods

**Reviewer:** 2. (line 40) You study about unmet need for family planning while you concluded about family planning, since unmet need is not equal with family planning

**Response:** Thank you for the observation. It has been corrected to read “prevalence of unmet needs for family planning was …’

**Introduction**

**Reviewer:** 1. It is good while some grammatical errors there and

**Response:** Thank you very much. Grammatical errors and other typographical errors have been corrected.

**Reviewer:** 2. There is also study conducted on unmet need for family planning while you did not mentioned it (Solanke et al.2022, adebowale et al,2023 based on 2018 DHS…)

**Response:** Thank you. However, Solanke et al. (2022), Adebowale et al. (2023) were cited.

**Reviewer:** 3. Since DHS data is open access you did not assure it is whether conducted or not similar study

**Response:** The DHS data is open access. We have mentioned that access was sought form the organisation before the use of their data. The DHS data set is in the public domain.

**Reviewer:** 4. There is study conducted based on Nigeria DHS 2018 which is recent data and important for policy and program, if this study conducted what is the important of studying on same title?

**Response:** Thank you. There is no studying having the same title known to us in literature using a pooled NDHS datasets 2008, 2013 and 2018. The study has contributed to the body of knowledge by providing information on changes and determinants of unmet needs for family planning

Reviewer: 5. Trends of unmet need for family services among conducted by Oginni et al. 2015 (based on DHS 2008-2013) and Adebowale et al. 2023 (based on 2018 DHS) if so what new finding is added this study?

Response: Thank you. The referred studies are not the same as this study. Oginni et al. 2015 study was based on DHS 2003, 2008 and 2013, and the study focused on trend and determinants of unmet need for family planning among currently married and sexually active unmarried women age 15-49 in Nigeria. However, this study is on changes and determinants of unmet needs for family planning among married women of reproductive age in Nigeria (based on DHS 2008, 2013 and 2018). Also, Adebowale et al. (2023) focused on unmet needs for family planning but specifically focused on high parity women of reproductive age using DHS 2018.

**Methods**

**Reviewer:** 1. How to manage missing values

**Response:** Thank you for the observation. Missing values and unknown were treated as missing in this study. This has been added.

**Reviewer:** 2. Detailed analysis of the data not described

**Response:** Thank you for the observation. As suggested by Reviewer #3, we agreed not to add more details than what we already had in this manuscript since the data is a secondary data which has been reported elsewhere. All detailed steps taken in the data analysis have been reported in the manuscript.

Reviewer: 3. Description of independent variables mention in tables

Response: Thank you for the observation. However, it is not compulsory that the description of independent variables should be mentioned using table

**Reviewer:** 4. What about clustering effect

**Response:** Thank you for the observation. Clustering effects have been taken care using multi-level regression model.

**Reviewer:** 5. Random effect and fixed effect, is two equally used how?

**Response:** Thank you for the observation. Both the random effect and fixed effect have been presented in Table 3, analysed and interpreted in the manuscript.

**Results**

**Reviewer** 1. What does mean Univariate Results? In table descriptive statistics/sociodemographic characteristic

**Response:** Thank you for the observation. Mean age and standard deviation were reported for age in single year and not on age group.

**Reviewer** 2. Missing value not reported, why? Since this is secondary data missed data is expected

**Response:** Thank you for the observation. It has been declared in the analysis as missing values and excluded from the analysis.

**Reviewer** 3. You use fixed effect model, how? This is country level data so how fixed effect model used?

**Response:** Thank you for the observation. The fixed/random effects model can be used as an alternative to traditional multilevel models to investigate the effects of individual-level variables as well as country-level variables.

**Reviewer:** 4. The ICC value decreased what does mean?

**Response:** Thank you for the observation. The ICC is an important tool for cluster-randomized pragmatic trials. ICC value show the proportion of the variance explained by the grouping structure in the population. Thus, a decreasing ICC implies ---------------------

**Discussion**

**Reviewer:** 1. The implication not included please include in discussion

**Response:** The implication of the study has been addressed.

**Reviewer:** 2. Comparison with previous literatures not well discussed

**Response:** The writers made use of the available literature on this subject and related it to the findings of this study. Plausible reasons were cited for the divergence and convergence of the findings.

**Reviewer:** 3. Include national level reports of unmet need during discussion

**Response:** When you mean national level reports of unmet needs, I believe the NDHS dataset used is a typical national level report for unmet. However, a report has been given in line with the NDHS dataset. This can be checked at line number 384 to 393.

**Reviewer:** 4. Religion is one of the factors, how did you recommended for programs and policy makers

**Response:** This has been addressed under the conclusion section.

**Reviewer:** 5. You calculated Odds ratio but on discussion it says there is a positive, how?

**Response:** Thank you for this observation. Misuse of ‘positive’ or ‘negative’ has been corrected.

**Limitation**

**Reviewer:** What about missed value, using secondary data, behavioral factors?

**Response:** Thank you for the observation. Missed values have been declared in the data analysis as missing values and excluded from the analysis. It has also been addressed under the strength and limitation section. Check at line number 473.

****************************************************************************

**REVIEWER #3**

**Abstract**

**Reviewer** (page 1, line 19 and 21). The objective states that the study focused on unmet need of reproductive women in Nigeria. However, under the "Material and Method" section in the abstract, it was written that the study examined the trend of unmet need. This is confusing. Kindly state clearly the objective of the study to enable readers follow through.

**Response:** Thank you for the observation. It has been corrected to read “Methods”. Also, the study objectives have been clearly stated to read “the study examined changes and contextual determinants of unmet need for family planning in Nigeria using pooled data.

**Introduction**

**Reviewer:** The study does not show the gaps in knowledge that necessitate the present study. The authors stated that between 2015 and 2018, an estimated 210 million pregnancies occurred. Kindly state the source of this information. Paragraph 3 and 4 is clumsy and not coherent. The sentences in the introduction are too long without precision. Please use short sentence. There is no clear direction of the problem in the introduction as merely outlining statistics does not imply problem. Kindly show the pain that gave birth to the study. You could commence from the global scale to the region (Africa) and national (Nigeria). The introduction does not flow systematically.

**Response:**	The research gap has been clearly stated in the introductory session. This is the first study to the best of our knowledge which examined the changes and contextual determinants of unmet need for family planning among women of reproductive age in Nigeria. Previous studies in Nigeria focused on different segments of the population and largely overlooked contextual determinants. We have revised the introduction substantially**.** In fact, it was entire re-written. First, the length has been reduced drastically. There is now a clear transition between sentences and paragraphs, reducing disjointed presentation of ideas. Each paragraph now connects smoothly to the next to maintain the flow of information. We have merged or rephrased some sentences (including the referent ones noted by the reviewers) for better coherence and readability.

**Method**

**Reviewer:** This is a standard secondary data and most of the details outline were not needed. Moreso, the authors stated similar studies were the method and dataset had been used. Brevity is power

**Response:** Thank you for the observation. Briefness we agreed is the power. As suggested, it has been corrected.

Result

**Reviewer**: State the prevalence of unmet need per cohort of DHS. What is responsible for variation of the prevalence?

**Response:** Thank you for the observation. Prevalence of unmet need per cohort of DHS as well as factors responsible for the variations have been included.

**Discussion**

**Reviewer:** summarise key result with reference to the objective of the study

**Response: The key results have been discussed by comparing it with prior studies and stating plausible reasons for the divergence and convergence of results.**

**Reviewer:** The context of the result is missing. This is expected based on the objective of the study stated at the abstract section

**Response: The discussion, based on the objectives of the study, has been contextualised. Plausible reasons cited for the patterns of results were gleaned from local literature and discussed in line with the settings of results.**

**Reviewer:** Can this result be generalise? Please state reasons

**Response: This result can be generalised because it used a standardized secondary data which used similar methodologies across countries. The data is nationally representative.**

**Reviewer:** What is the policy implication of the study?

**Response: The policy implications have been added in each of the objectives. The overall policy implication is that policy makers and programmes should take not of the slip in the decline in unmet need for family planning within the referenced years and consider the contextual determinants identified to be responsible for these changes in future interventions.**

**Reviewer:** Argument in this section is not coherent

**Response: The discussion section has been improved substantially by making the arguments coherent, comparing and contrasting plausible reasons for the patterns of results**

**Conclusion**

**Reviewer:** Where is the novelty of the study?

**Response: This current study to the best of our knowledge is the first to examine changes and contextual determinants of unmet need for family planning among women of reproductive age in Nigeria. The oscillatory decline and increase in unmet need for family planning in the referenced period is an important pointer for policy makers and programmers to design interventions which would address the slip in decline based on the significant contextual factors associated with the changes in this study.**
